# Cerium Oxide Nanoparticles (Nanoceria): Hopes in Soft Tissue Engineering

**DOI:** 10.3390/molecules25194559

**Published:** 2020-10-06

**Authors:** Hossein Sadidi, Sara Hooshmand, Ali Ahmadabadi, Seyed Javad Hoseini, Francesco Baino, Morvarid Vatanpour, Saeid Kargozar

**Affiliations:** 1 General Surgery Department, Ghaem Hospital, Mashhad University of Medical Sciences, Mashhad 9176999311, Iran; h100ze@gmail.com; 2 Pharmacological Research Center of Medicinal Plants, Mashhad University of Medical Sciences, Mashhad 917794-8564, Iran; s_hooshmand@yahoo.com; 3 Department of Pharmacology, Faculty of Medicine, Mashhad University of Medical Sciences, Mashhad 917794-8564, Iran; 4 Surgical Oncology Research Center, Mashhad University of Medical Sciences, Mashhad 9176999311, Iran; ahmadabadia@mums.ac.ir; 5 Department of Medical Biotechnology and Nanotechnology, Faculty of Medicine,, Mashhad University of Medical Sciences, Mashhad 917794-8564, Iran; hoseinij@mums.ac.ir; 6 Institute of Materials Physics and Engineering, Applied Science and Technology Department, Politecnico di Torino, Corso Duca degli Abruzzi 24, 10129 Torino, Italy; 7 Department of Anatomy and Cell Biology, School of Medicine, Mashhad University of Medical Sciences, Mashhad 917794-8564, Iran; morvarid.vatanpour75@gmail.com; 8 Tissue Engineering Research Group (TERG), Department of Anatomy and Cell Biology, School of Medicine, Mashhad University of Medical Sciences, Mashhad 917794-8564, Iran

**Keywords:** biomaterials, cerium oxide nanoparticles, nanoceria, skin wounds, nervous system, cardiac regeneration, ophthalmology, soft tissue engineering

## Abstract

Several biocompatible materials have been applied for managing soft tissue lesions; cerium oxide nanoparticles (CNPs, or nanoceria) are among the most promising candidates due to their outstanding properties, including antioxidant, anti-inflammatory, antibacterial, and angiogenic activities. Much attention should be paid to the physical properties of nanoceria, since most of its biological characteristics are directly determined by some of these relevant parameters, including the particle size and shape. Nanoceria, either in bare or functionalized forms, showed the excellent capability of accelerating the healing process of both acute and chronic wounds. The skin, heart, nervous system, and ophthalmic tissues are the main targets of nanoceria-based therapies, and the other soft tissues may also be evaluated in upcoming experimental studies. For the repair and regeneration of soft tissue damage and defects, nanoceria-incorporated film, hydrogel, and nanofibrous scaffolds have been proven to be highly suitable replacements with satisfactory outcomes. Still, some concerns have remained regarding the long-term effects of nanoceria administration for human tissues and organs, such as its clearance from the vital organs. Moreover, looking at the future, it seems necessary to design and develop three-dimensional (3D) printed scaffolds containing nanoceria for possible use in the concepts of personalized medicine.

## 1. Introduction

The role of metals in humans’ daily lives is notable, and several metallic elements are currently being used to diagnose and treat a broad range of injuries and diseases [1,2]. With the emergence of nanotechnology, smaller sizes of metals (1-100 nm) were evaluated by scientists to exactly determine their advantages and disadvantages for living systems, as nano-sized particles exhibit distinct biological effects in comparison to their bulk counterparts [3,4]. Among different types of nano-metals, cerium oxide nanoparticles (CNPs or nanoceria) are considered one of the most promising substances in biomedical engineering approaches [5]. 

Cerium (Ce) is one of the most abundant rare-earth metals categorized as a member of the lanthanide series in the periodic table [6]. In the bulk state, Ce can be found in dual oxidation modes (Ce^3+^ and Ce^4+^) and two distinct redox states, resulting in the formation of oxides named cerium dioxide (CeO_2_) and cerium sesquioxide (Ce_2_O_3_) [7]. As compared to Ce^3+^, the oxidation state Ce^4+^ is more stable in nature thanks to its electronic structure. However, ceria at the nanoscale form possesses a mixture of the 3+ and 4+ states on its surfaces [8]. It should be mentioned that the number of 3+ sites increases along with the decrease of the particle diameter, leading to losing oxygen atoms (oxygen vacancies) on the surface of CNPs [9,10]. The Ce^4+^/Ce^3+^ redox cycle renders CNPs a multienzyme mimetic activity by which free-radical scavenging, radiation protection, and oxidative stress attenuation are performed [11,12,13]. These properties make CNPs potent substances in tissue engineering and regenerative medicine applications [14]. However, the toxic effects of CNPs in the biological systems should be carefully considered; for example, programmed cell death (apoptosis) and autophagy have been reported in human peripheral blood monocytes after the exposure to nanoceria under certain conditions [15]. 

From the tissue engineering point of view, CNPs offer prominent biological features for accelerating tissue repair and regeneration, including antioxidant, anti-inflammatory, antibacterial, angiogenic, and antiapoptotic activities [16,17,18,19,20]. There are huge numbers of experimental studies clarifying the therapeutic effectiveness of CNPs, either in bare or functionalized forms for biomedical applications [19]. For example, samarium ion-doped CeO_2_ (SmCeO_2_) nanoparticles conjugated with (6-(2-[2-(2-methoxy-ethoxy)-ethoxy]-ethoxy)-hexyl) triethoxysilane moieties can improve endothelial cells (ECs) proliferation, overexpress proangiogenic markers (p38 MAPK/HIF-1α), and induce neovascularization in a chick embryo [21]. Incorporating CNPs into polymeric matrices is also a common method to make composites for diverse tissue engineering applications, such as accelerated excisional wound healing [22]. Moreover, three-dimensional (3D) engineered scaffolds containing CNPs were found to be suitable substitutes for replacing damaged skin tissue [23,24]. As the application of hard materials in soft tissue engineering brings new hope in the concept of modern therapies, we tried to clearly show the pros and cons of CNPs in reconstructive strategies applied for the skin, cardiac, neural, and ophthalmic tissues. To the best of the authors’ knowledge, this is the first report that specifically focuses on the potential of CNPs in managing damages and diseases of various soft tissues.

## 2. Wound Healing: What We Know and Need

**Figure 1:**
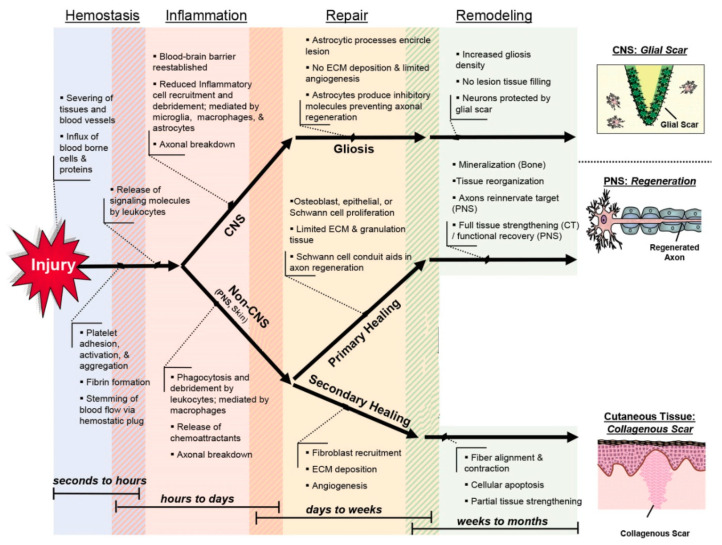
Schematic illustration of the wound-healing process in soft tissues post-injury. Reproduced with some modifications from [26].

After being injured, human tissues undertake a series of molecular and cellular events to rebuild and recover their structure and function at injured sites. It has been previously stated that wound healing has an almost similar trend in different tissues (e.g., the skin and the nervous system) [25]. The healing process is usually categorized into four highly integrated and overlapping stages, including hemostasis, inflammation, proliferation, and remodeling (see Figure 1) [26]. The wound-healing process takes place over a variable time range lasting up to several days post-injury, and its duration depends on some parameters like the extent of the lesion, the type of tissue, and the overall patient’s health condition [27]. The primary cells involved in the healing process include platelets, neutrophils, macrophages, and fibroblasts [28]. These cells secrete a range of cytokines and growth factors, including interleukins (ILs) and tumor necrosis factor alpha (TNF-α), as well as a platelet-derived growth factor (PDGF), to recruit white cells and fibroblasts and stimulate fibroblast and keratinocyte proliferation. Matrix metalloproteinases (MMPs) and their inhibitors, i.e., tissue inhibitors of metalloproteinases (TIMPs), are other essential effectors playing pivotal roles in the wound-healing process; they can attenuate inflammation, promote re-epithelialization, and regulate the remodeling [29]. 

Delayed acute and chronic wounds (impaired healing) usually happen when the physiologic wound repair fails to progress through the normal stages. Extreme near-anoxic hypoxia, infection, stress, diabetes, obesity, alcoholism, and smoking are the major risk factors for creating nonhealing chronic wounds [27]. Generally, no signs of effective healing are observed in the chronic wounds within three months post-injury; on the contrary, there is a prolonged inflammatory phase (high levels of IL1 and IL6), overabundant neutrophil infiltration, and continuous infections [30,31,32]. In order to prevent these complications, huge numbers of substances, techniques, and technologies have been developed and used in the clinical setting [33,34,35]. Among the various materials applied in treating soft-tissue wounds, nanoceria has shown great promises during both in vitro and in vivo experiments [36,37,38]. In Section 2, the benefits of nanoceria for soft-tissue repair and regeneration will be introduced and discussed. 

## 3. Nanoceria: Biological Superiorities for Soft-Tissue Healing

### 3.1. Cell and Tissue Compatibility

One of the most critical parameters for any substances administrated into the human body is the compatibility with the cells and tissues. There are several studies in the literature presenting nanoceria as a bio-safe substance [39,40]; however, some in vitro and in vivo research reported levels of acute and chronic toxicity for it [41,42,43]. As an illustration, CeO_2_ nanoparticles were recently reported as genotoxic substances for human peripheral blood cells at concentrations of 0.78, 1.56, 3.125, 6.25, 12.5, 25, and 50 ppm within 72 h under in vitro conditions [44]. 

Some parameters were identified as effective factors in the toxicity of nanoceria, including the synthesis method, particle size and shape, surface charge, and cell type, as well as the dose and exposure route [45,46,47]. For instance, environmentally relevant concentrations of nanoceria (dose range 6 × 10^−5^–6 × 10^−3^ g/L, corresponding to a concentration range of 0.22–22 µM) caused DNA damages to primary human dermal fibroblasts [48]. In general, nanoceria may cause toxicity via similar mechanisms induced by other low-soluble nano-sized materials, as well as through its unique surface chemical nature [49]. The main mechanisms proposed for nanoceria toxicity could be summarized as (I) activating oxidative stress-independent mechanisms in case of high aspect ratio nanorods and nanowires (≥ 22) [50], (II) changing the surface valence state configuration in contact with biological molecules, resulting in a significant decrease of Ce^4+^ and the presence of Ce^3+^ on the surface (more redox active state), with subsequent genotoxicity [51], and (III) inducing the production of reactive oxygen species (OH^•^) in the case of persistent long-term exposure (see Equation (1)). Equation (1):

(I) Ce^4+^ + A^−^_red_ → Ce^3+^ + A_ox_ (II) Ce^3+^ + O_2_ → Ce^4+^ + O_2_^−^ (III) O_2_^−^+ O_2_^−^ → O_2_ + H_2_O_2_ (IV) H_2_O_2_ + Ce^3+^ → Ce^4+^ + OH^−^ + OH^•^ (V) LOOH + Ce^3+^ → Ce^4+^+LO^•^ + OH^−^ (1)

where A^−^_red_ is a physiologically relevant reductant (e.g., thiol or ascorbate), and LOOH is lipid peroxide. As observed in Equation (1) (IV), Ce^3+^ via its catalase mimetic activity breaks down hydrogen peroxide (H_2_O_2_) and creates a reactive oxygen species (hydroxyl, OH^•^). Similarly, it may break down lipid hydroperoxides (LOOHs) to lipid peroxyl radicals (LO^•^), implying destructive effects on mammalian cells. 

On the other hand, there are plenty of experiments reporting interesting strategies to improve nanoceria’s cyto- and tissue-compatibility [52,53]. The coating of CeO_2_ nanoparticles by other materials (e.g., dextran and carbon) has been reported as an effective method for lowering nanoceria’s nondesirable effects in the long term [54,55]. Regarding soft-tissue engineering applications, nanoceria’s toxic effects were well-studied both in vitro and in vivo. For example, it has been reported that 5 and 10 μg/mL of CeO_2_ has no genotoxic effect on human lens epithelial cells [56]. Furthermore, nanoceria at dosages of 500 nM, 1 μM, and 10 μM could enhance the proliferation and migration of fibroblasts, keratinocytes, and vascular endothelial cells (VECs) [57]. It is worth mentioning that the use of nanoceria for ameliorating doxorubicin-induced cardiotoxicity was quite effective [58]. 

### 3.2. Antioxidant, Antiapoptosis, and Anti-Inflammatory Activities

Based on the definition, oxidative stress represents an imbalance between the generation and elimination of reactive oxygen species (ROS). ROS act as signaling mediators regulating many cellular activities, including growth, proliferation, differentiation, apoptosis, and autophagy [59,60]. However, increased ROS levels may lead to creating oxidative stress and consequent pathogenic conditions [61]. To date, various antioxidant agents have been applied to manage ROS levels; nanoceria is among the most promising candidates in soft tissue applications [16,62]. CeO_2_ nanoparticles showed excellent antioxidant enzyme-mimetic activity and the potential of scavenging not only ROS (e.g., superoxide radical anion (O_2_^•−^)) but, also, reactive nitrogen species (RNS) (e.g., nitric oxide radical (^•^NO)) [63]. However, nanoceria’s antioxidant properties are dose- and pH-dependent; it may generate and stabilize ROS at high concentrations and acidic conditions while attenuating free radicals at low concentrations and neutral pH [64]. Antioxidant enzyme-mimetic activity and antioxidant ROS/RNS scavenging activity of CNPs are shown in Equation (2) (I-III) and (IV-VI), respectively [65]: Equation (2):

(I) O_2_^•−^ + Ce^3+^ + 2H^+^ → H_2_O_2_ + Ce^4+^ (Ce^3+^ is oxidized) (II) O_2_^•−^ + Ce^4+^ → O_2_ + Ce^3+^ (Ce^4+^ is reduced) (III) H_2_O_2_ + 2Ce^4+^ + 2OH^−^ → 2H_2_O + O_2_ + 2Ce^3+^ (CNPs with low Ce^3+/^Ce^4+^ surface ratios) (IV) Ce_2_O_3_ + 2[^•^OH] → 2CeO_2_ + H_2_O (V) 2CeO_2_ (in presence of aqueous H^+^) → Ce_2_O_3_ + ½O_2_ (VI) Ce^4+^ + ^•^NO → [Ce^4+^ + NO ↔ Ce^3+^ + NO^+^] (CNPs with low Ce^3+^/Ce^4+^ surface ratios) (2)

It has been well-shown that CNPs can absorb and release oxygen plus efficient redox cycling between Ce^3+^ and Ce^4+^ on their own surface, performing scavenging ROS and RNS [66,67]. Molecular mechanisms behind the antioxidant effects of CeO_2_ nanoparticles were recently reviewed [68]: Nuclear factor (erythroid-derived 2)-like 2 (Nrf2) and nuclear factor-κB (NF-κB) transcription factors, as well as the MAPKs and PI3K-AKT pathways, are the main targets for nanoceria, implying their antioxidant activity [69,70]. In 2018, Rather et al. successfully developed CNP-functionalized poly(ε-caprolactone) (PCL)-gelatin electrospun fibers for skin wound-healing applications [71]. They found that the crystallinity of the oriented nanofibers was ∼2.6 times lower than the pristine counterparts, resulting in the rapid degradation of nanofibers and the release of CNPs. The CNP-incorporated mats showed superoxide dismutase (SOD)-mimetic activity and enhanced fibroblast proliferation up to ∼48% in comparison to the controls. The authors concluded that this nanocomposite might be a promising candidate for in vivo wound-healing strategies. 

Huge numbers of experimental data showed the protecting effects of nanoceria against apoptosis induced by oxidative stress [72,73,74,75]. Indeed, nanoceria’s intracellular antioxidant effect is the direct basis of their antiapoptotic and pro-survival effects, and all these outstanding features depend on Ce^3+^/Ce^4+^ redox reactions [76]. Stabilizing the mitochondrial membrane potential in mammalian cells was also stated as another mechanism behind nanoceria’s antiapoptotic activity [77]. 

Previous studies have shown that nanoceria does not evoke inflammatory reactions, even at high concentrations (e.g., 50 μg/mL) [78]. On the other hand, some experimental data indicate the potential of CNPs in attenuating inflammation both in vitro and in vivo [79,80]. CNPs showed the ability to downregulate the gene expression of proinflammatory cytokines (thymic stromal lymphopoietin (TSLP) and leukemia inhibitory factor (LIF), as well as interleukins 3 and 7 (IL3 and IL7)) while upregulating anti-inflammatory IL-6 and IL-13 [81,82]. As ROS play a central role in the progression of inflammation, the ROS scavenging ability of CNPs is considered another mechanism behind their anti-inflammatory effects [83]. 

### 3.3. Angiogenic Activity

**Figure 2:**
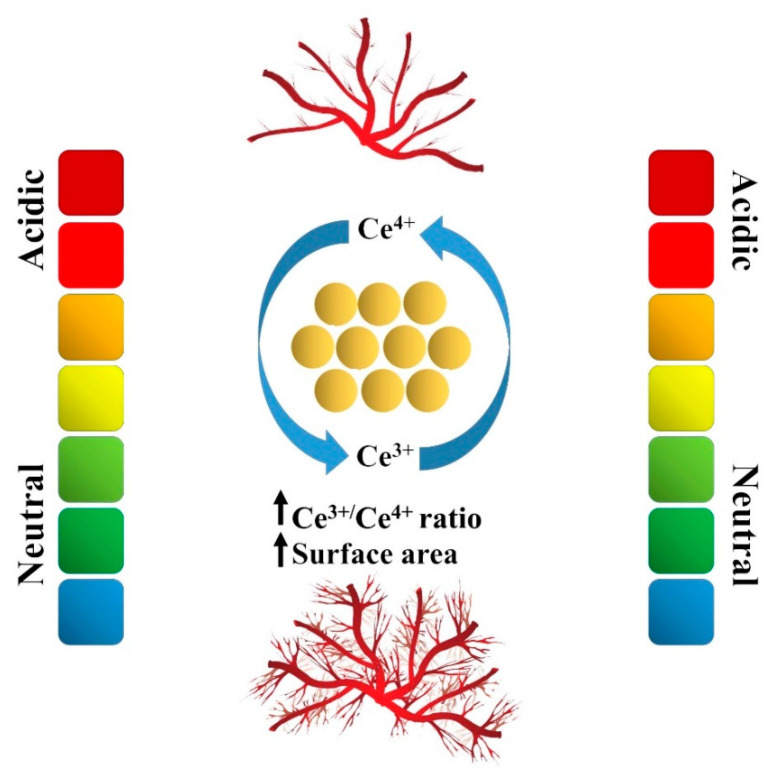
Schematic representation showing the impact of the environment on the angiogenic capacity of cerium oxide nanoparticles (CNPs). The environmental pH, reactive oxygen species (ROS) generation, and intracellular oxygen concentration determine the pro- or antiangiogenic behavior of CNPs. Reproduced from [86].

Experimental evidence indicates the ability of CNPs in both inducing and inhibiting neo-vessel formations (Figure 2). A few parameters have been identified as controlling elements in determining the pro- or antiangiogenic activity of CNPs; the microenvironment conditions (e.g., pH) and applied dosages are among the major determinants [84]. For illustration, high concentrations (> 8.6 mg/mL) of nanoceria may hinder the proliferation of human umbilical vein endothelial cells (HUVECs) [85].

Regarding proangiogenic applications, a high surface area and increased Ce^3+^/Ce^4+^ ratio are recognized as key factors in making nanoceria a robust inducer of angiogenesis [87]. Additionally, some reports emphasized the critical impact of shape and size of CNPs on the outcomes. Nanoparticles or nanostars did not elicit toxic effects in HUVECs, whereas ceria nanorods caused a slight decrease in EC proliferation [87]. The ceria particles with a size <15 nm showed the ability to induce tubule formation, while micrometer-sized ones prohibited the tube formation in HUVECs [87]. The stabilization of hypoxia-inducible factor 1-alpha (HIF1-α) in ECs and upregulated vascular endothelial growth factor (VEGF) expression were stated as mechanisms affected by CNPs [87]. It should be mentioned that other studies introduced nanoceria as an activator of the p38-MAPK/IF-1 α signaling pathway and, subsequently, promoted angiogenesis [21].

### 3.4. Antibacterial Properties

**Figure 3:**
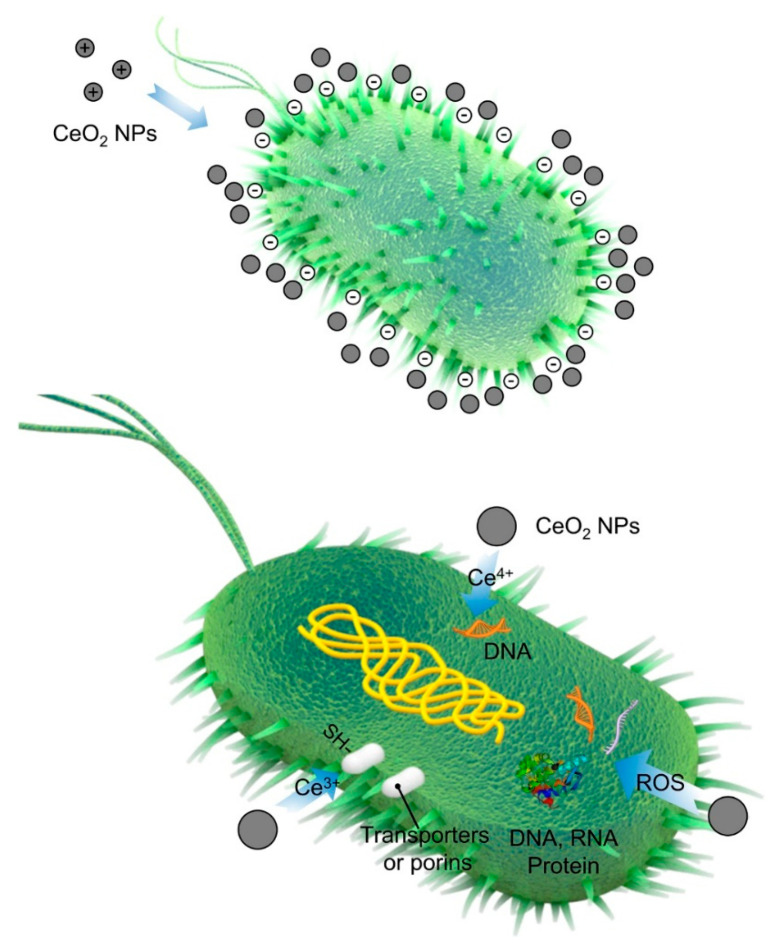
The representation of the main antibacterial mechanism of CNP-based materials. Reproduced from [88].

The antibacterial properties of CNPs were recently well-reviewed by Qi et al. [88]. The potential of nanoceria for either inhibiting or killing Gram-positive and Gram-negative bacteria has been identified through well-identified mechanisms; the direct contact between CNPs and bacterial membranes is considered the main factor [89]. In this regard, positively charged CeO_2_ nanoparticles adsorb onto negatively charged membranes of bacteria by electrostatic interactions and persistently remain on their surfaces. This leads to a change of the membrane viscosity, impairment of the specific ionic pumps, and the subsequent disturbance of bacterial growth in the long term. Increasing the ROS levels and consequent damages to DNA, RNA, proteins, polysaccharides, and lipids is another destructive effect of nanoceria on bacterial cells [90]. The third mechanism is related to the attack of nanoceria to the outer membrane of the bacteria, which results in altering the electron flow and respiration of bacteria and, consequently, hampering the nutrient transportation (cellular respiration, oxygen uptake, and glucose metabolism) (see Figure 3) [91,92]. 

It may be interesting to mention that nanoceria have also been proposed as a potential antibiotic adjuvant. CNPs showed the ability to enhance the outer membrane permeability coefficient in *Escherichia coli* (*E. coli*) and the antibacterial activity of beta-lactam antibiotics against *Klebsiella pneumonia* (*K. pneumonia*) [93].

As polymeric constructs provide suitable soft-tissue replacements, CNP-incorporated nanofibers, films, and hydrogels were developed and used for managing infectious wounds [36,94,95]. More recently, chitosan and cellulose acetate composite films containing CNPs (0.1% (w/v) and 1% (w/v)) were prepared as potential wound dressings [96]. The inhibitory effects of the CNP-containing films against *Staphylococcus aureus* (*S. aureus*) and *E. coli* bacteria proved their antibacterial activity; increasing CNP amounts in the films resulted in considerable antibacterial activity.

## 4. Nanoceria for Soft-Tissue Engineering

### 4.1. Skin Wound Healing

Up to now, different types of natural and synthetic substances have been developed and applied for managing skin wounds; all could be categorized into three groups, including bioactive, interactive, and passive [97,98]. CNPs as bioactive materials have been introduced as remarkable therapeutics for skin tissue repair and regeneration [99,100]. Nanoceria is recognized as a stimulator of fibroblast proliferation in vitro [101,102] and an accelerator of the healing of model lesions in vivo [103,104]. Having unique antioxidant, anti-inflammatory, and antibacterial properties, CNPs have attracted much attention for use in the treatment of both acute (e.g., burns) and chronic (diabetic wounds) skin lesions. 

The use of CNPs for managing chronic wounds caused by diabetes has been promising regarding their ability to prevent infections and accelerate wound closure [105]. It has been demonstrated that the administration of varying concentrations of nanoceria-miR-146a (1, 10, 100, and 1000 ng) may be effective in treating diabetic wounds created on the dorsal skin of Db/Db mice. Although the treatment with 10, 100, or 1000 ng of nanoceria-miR-146a was effective in wound healing within 14 days post-administration, a 100-ng dosage was found as the optimum concentration of the nano-dressing [106]. 

**Figure 4:**
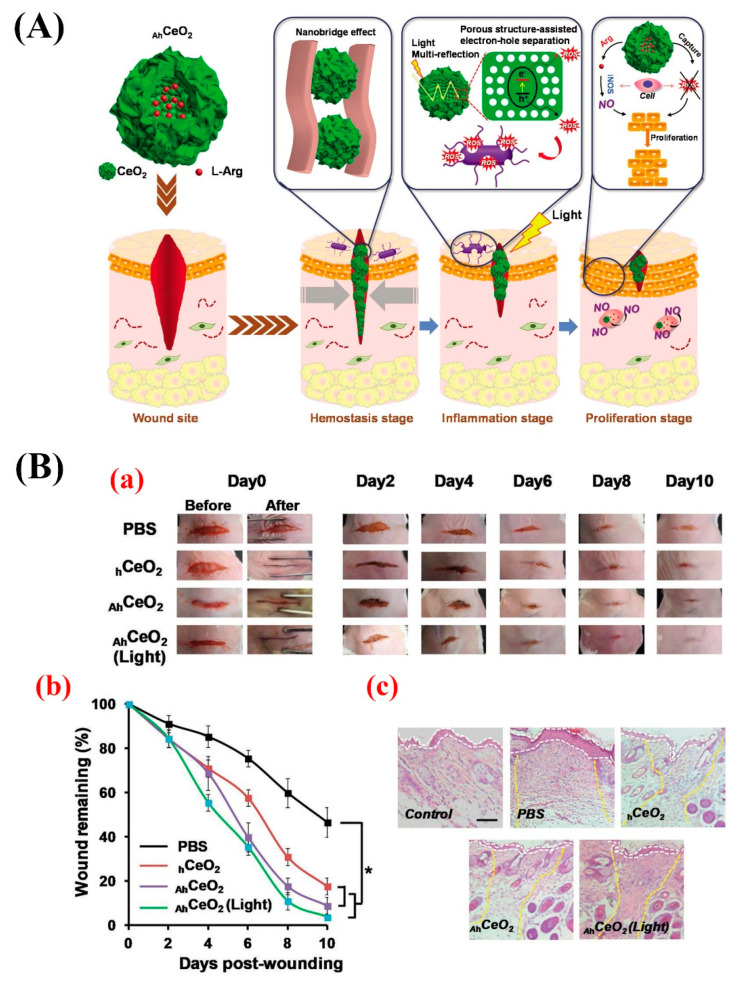
(**A**) Schematic representation of the hierarchical promotion of multiple wound-healing stages by applying a rough surface on L-arginine (L-Arg) inside CNPs (_Ah_CeO_2_NPs). At the hemostasis stage, the modified nanoparticles can serve as a tissue nanobridge to rapidly close the wound and inhibit the bleeding. At the inflammatory stage, _Ah_CeO_2_ NPs show excellent light-harvest efficiency based on light multi-reflection properties inside hollow structures, as well as high electron-hole separation efficiency thanks to their porous shell, which can efficiently produce reactive oxygen species (ROS) to kill bacteria under simulated sunlight irradiation. At the proliferation stage, the particles can capture the excess ROS generated at the wound site due to their superoxide dismutase (SOD) and catalase (CAT) activities, and the released L-Arg can be converted into nitric oxide (NO) by inducible nitric oxide synthase (iNOS) overexpressed in macrophages and, subsequently, promoting cell proliferation. (**B**) Micrographs showing the wound healing within 10 days post-treatment, (a) the quantification of wound-healing kinetics expressed as a percentage of the initial wound length, (b) and hematoxylin and eosin (H&E)-stained tissue samples at 10 days post-treatment (c) (the white dashed lines indicate the boundary of the epidermal layer, and the yellow dashed lines show that the boundary of the wound area remains fully regenerated). Scale bar: 100 µm. Reproduce from [107].

In 2019, Ma et al. developed hollow CNPs (size 104.3 ± 13.1 nm) with a rough surface and incorporated L-arginine inside them to provide a compact and programmable nanosystem for sequentially promoting multiple stages of wound healing (the hemostasis, inflammation, and proliferation stages) (Figure 4A,B) [107]. The authors showed that the rough surface of this system could act as a nanobridge for fast wound closure in mice, leading to the promoted structural recovery of the injured skin and, subsequently, accelerating the hemostasis stage. The nanosystem could also generate abundant ROS for bacteria inactivation as a result of the light multireflection inside the hollow structure and porous shell of the nanocomposite. This led to promoting wound-healing during the inflammation stage by preventing wound infection. The SOD- and catalase (CAT) activity-mimicking feature of the nanosystem could alleviate the oxidative damage in the wound site and, eventually, promoted epithelial cell proliferation (the proliferation stage). 

To date, several CNP-containing materials, including polymeric constructs, ceramics, and different composites, have been proven to have extensive potential for wound healing [71,108,109]. For example, nanoceria-functionalized polycaprolactone-gelatin electrospun nanofibers were prepared and used to enhance wound healing. This nanocomposite showed the ability to enhance the proliferation of 3T3-L1 cells by 48% and exhibited a SOD-mimetic activity due to the presence of CNPs in the composite [71]. In another study, nanoceria (1% *w/w*) was added to electrospun poly(3-hydroxybutyrate-co-3-hydroxyvalerate) (PHBV) membranes to enhance the cell adhesion and proliferation, as well as angiogenesis, in diabetic wound sites [110]. According to the results, nanoceria membranes (<1% *w/w* of nanoceria content) could promote cell adhesion, proliferation, and vascularization when applied as wound dressings. 

Recently, Bhattacharya et al. took advantage of the synergic therapeutic effects of CNPs and curcumin and prepared a polyacrylamide hydrogel containing these substances to make antioxidant and anti-inflammatory wound dressings [111]. They showed that the CNP- and curcumin-incorporated scaffolds may lead to higher wound-healing efficacy (78%) and negligible scarring in a full-thickness acute wound-healing model of rats as compared to dressings containing only curcumin or CNPs in seven days. Monocyte chemoattractant protein 1 (MCP-1) and transforming growth factor beta (TGF-β) were suggested to be the biomolecular factors involved in accelerated healing. Furthermore, the authors stated that the promoted almost-scarless healing was attributed to the upregulation of the growth-related signaling pathways of HER2/ErbB2, TGF-β-Smad2/3, MAPK/ERK, AKT, and VEGF. 

In addition to polymer-based constructs, CNPs were also used in combination with other biomaterials; ceria nanocrystal-decorated mesoporous silica nanoparticles (MSNs) were successfully prepared to serve as ROS scavenging tissue adhesive agents and applied for the regeneration of cutaneous wounds of rats. The application of this system could bring the wound edges together and thereby facilitate the restoration of the tissue barrier function. Furthermore, the ROS scavenging tissue adhesive could provide a friendly microenvironment for skin regeneration via alleviating oxidative stress in the injured site.

In order to sum up, the use of CNPs for managing chronic skin wounds was quite effective, and there are some clinical trials for proofing this issue [112]. However, limited research exists over the applicability of CNPs for treating acute wounds like burns, and this is may be an interesting area for future in vitro and in vivo experiments. In addition, designing and developing novel formulations of CNP-containing glass and glass ceramics should be considered for skin wound healing. Based on prior published data [113,114], CNPs may be useful for preventing and treating malignant skin wounds, either alone or in combination with other materials. However, the toxicity and the removal procedure of CNPs from damaged sites should be well-identified after their administration.

### 4.2. Regeneration of the Nervous System 

Nanomaterials with the ability to control cell-material interactions have shown unique promise in treating nervous system disorders. Among various applied nanomaterials, nanoceria is recognized as a potent remedy for different parts of the nervous system, such as peripheral nerves. In this regard, the in vitro and in vivo cytoprotective and antioxidant effects of nanoceria were mentioned as its major advantages [115]. However, some concerns exist over CNP developmental neurotoxicity hazards [116]. 

Nanoceria could impact signal transduction pathways in neuroprotection and neuronal death in neurodegenerative disorders (e.g., Alzheimer’s and Parkinson’s diseases and ischemic stroke) due to its antioxidant properties and reduce the damage to normal cells [117]. The harmful effects of ROS, an important factor in disease pathogenesis, can lead to oxidative damage to DNA and proteins, lipid peroxidation, and cell death. In this regard, nanoceria can alleviate these destructive events based on its therapeutic properties, including anti-inflammatory and antiapoptotic activity, ROS removal capacity, and protein kinases inhibition. Toward taking the therapeutic effects of nanoceria in clinical nanomedicine, it is essential to consider the pathophysiology of target diseases, particularly for brain diseases, such as intracerebral and subarachnoid hemorrhage, Alzheimer’s and Parkinson’s syndromes, and ischemic stroke [118]. 

The use of neural stem cells transplantation has become of significant attention in managing neurodegenerative diseases associated with cognitive failure. The present approaches to promote neurogenesis are mostly based on the majorization of neural stem cell niche components and the directional differentiation of neurons. However, the high pathological level of oxidative stress damages the neurons derived from neural stem cells during treatment and compromises the neurogenesis effects. In order to overcome this drawback, an effective and simple methodology was reported by Yu et al. for modulating the neuron directional differentiation and ameliorating the oxidative stress [119]. They integrated antioxidative nanoceria nanozymes into metal-organic frameworks (MOF) to enhance neurogenesis synergistically. Small interfering RNA (siSOX9) and retinoic acid (RA) were also loaded in the MOF. The authors showed that the integration of nanoceria can accomplish strong SOD- and CAT-mimetic activities, resulting in eliminating ROS and avoiding oxidative damage to newborn neurons with subsequently extended survival rates and higher outgrowth of the newborn neurons. Due to the great drug delivery efficacy of MOFs and tremendous antioxidative capacity of ceria nanozymes, the designed nanosystem could extensively improve neurogenesis and promote the cognitive functions of an aged triple-transgenic Alzheimer’s disease (AD) model of mice. 

It has been well-documented that a spinal cord injury can aggravate the secondary injury, leading to a permanent, stable, functional impairment because of producing extra ROS in the damaged site. Nanoceria, due to its strong ROS scavenging effects, was selected as a suitable candidate to suppress inducible nitric oxide synthase (iNOS) generation and enhance the viability of H_2_O_2_-insulted cortical neurons [120]. CNPs at a dosage of 50-4000 μg/mL were administered to recovering contused spinal cord in rats, and the wound-healing progression was monitored during eight weeks post-injury. After one day, the iNOS-affected cell number in the treated groups was reduced compared to the control group. Furthermore, the inflammatory cells and cavity size were significantly decreased after seven days, and a downregulation in the expression of proinflammatory and apoptotic molecules and a synchronized upregulation of anti-inflammatory cytokine were recorded. By eight weeks, a considerably upgraded locomotor function was observed in treated groups compared to the control group. The effectiveness of nanoceria was also documented in other parts of the nervous system; the administration of CNPs in animals suffering from mild traumatic brain injury has led to improved therapeutic outcomes due to their ROS scavenging effects [121]. 

Polymers containing CNPs are also proposed excellent materials for managing different brain and spinal cord injuries [52]. For instance, cognition enhancing and neuroprotective activities of polyethylene glycol (PEG)-coated nanoceria (size 3 nm) were evaluated during hypobaric hypoxia in rat brains. PEG nanoceria could significantly decrease the oxidative stress and associated damages upon hypoxia exposure in the animals, as well as increase the hippocampus neuronal survival and stimulate neurogenesis [122]. 

**Figure 5:**
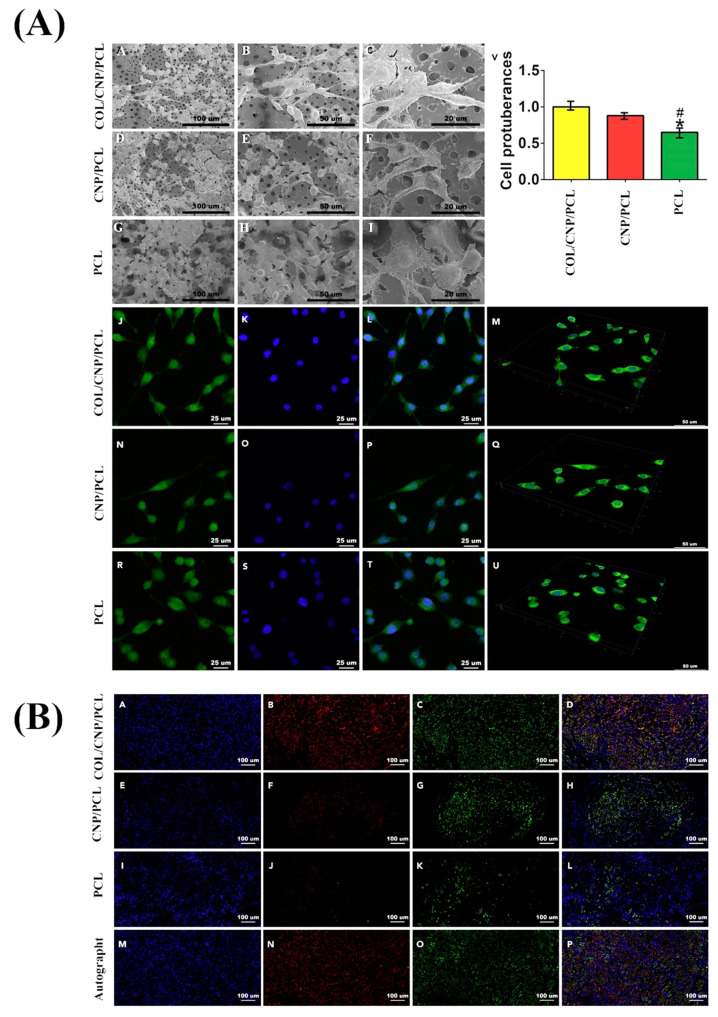
(**A**) Micrographs exhibiting Schwann cell morphology in different conduits captured by scanning electron microscope (SEM) (A–I) and immunofluorescence microscope (J–U). Note that the samples J, N, and R were stained by phalloidin; samples K, O, and S were stained by DAPI (4’, 6-diamidino-2-phenylindole, dihydrochloride); and samples L, P, and T are merged images; M, Q, and U are 3D displays for cell attachments in different conduits; and (V) showing cell protuberances in different conduits. (* *p* < 0.05 compared with collagen (COL)/ cerium oxide nanoparticles (CNP)/ polycaprolactone (PCL), # *p* < 0.05 compared with CNP/PCL). (**B**) Microscopic images exhibiting the triple immunofluorescence of regenerated nerves indicating axonal restoration after 18 weeks of the implantation: A, E, I, and M were stained by DAPI; B, F, J, and N were stained by NF200; C, G, K, and O were stained by Tuj1; and D, H, L, and P show merged images. Reproduced with permission from [127].

The use of nanocomposites has been quite effective for neurodegenerative disorders therapy [123,124,125]. In this regard, Marino et al. prepared highly aligned gelatin/nanoceria nanocomposite fibers by the electrospinning technique [126]. They showed that nanoceria, as a strong self-regenerative antioxidant, can behave as a powerful ROS scavenger to inhibit cell senescence and stimulate neurite sprouting. In 2019, Qian et al. evaluated the therapeutic effects of nanoceria in a 3D composite channel for treating a severe neurological defect [127]. To this end, they incorporated 0.5%, 1%, and 2%–4% nanoceria into a collagen/PCL (COL/PCL) blend to prepare composite conduits by asymmetrical 3D manufacturing. The in vitro assessments indicated that this scaffold improved the proliferation, adhesion, and neural expression of Schwann cells. Moreover, the in vivo implantation of the 3D conduits in a 15-mm rat sciatic nerve defect model confirmed the scaffold’s great potential in alleviating inflammation and oxidative stress and promoting angiogenesis. These positive biological phenomena contributed to the functional, electrophysiological, and morphological restoration of rat nerves at 6, 12, and 18 weeks post-implantation (see Figure 5). 

### 4.3. Cardiac Regeneration

Nanoceria has also gained growing attention for its potential use in managing various cardiac implications due to its antioxidative and anti-inflammatory effects [128]. For example, it may be an effective therapy for a myocardial reperfusion injury associated with abnormal enhancement in the ROS levels. CNPs were previously proven to act as a cardioprotective agent against cardiotoxic factors and could control the oxidative stress in cardiac cells [129]. Moreover, it has been reported that the intravenous administration of CNPs (15 nmol) in CP-1 transgenic mice (MCP mice) remarkably inhibited progressive left ventricular dysfunction and dilatation within two weeks [130]. 

Cardiac progenitor cells are known as a promising autologous source for cardiac regenerative medicine. However, suitable physicochemical and mechanostructural factors are of great importance for the in vitro culturing of cardiac progenitor cells to provide a complex array of bioactive substance concentrations, thoroughly mimicking their natural microenvironment in vivo [129,131]. Nanoceria could strongly control the oxidative stress in isolated cardiac progenitor cells, since it is a redox active substance. Pagliari et al. reported that 24-h exposure to nanoceria (5, 10, and 50 μg/mL) not only had no negative impact on cell function and growth in cardiac progenitor cells but, also, protected cardiac progenitor cells for at least seven days from H_2_O_2_-induced cytotoxicity [129].

It has been well-understood that microRNAs (miRNAs) and ROS are simultaneously involved in heart ischemia reperfusion injury due to their mutual cross-talks during the injury process [132]. In a study by Limin Yang et al., a novel crown-like silica-polydopamine/DNA/nanoceria composite was developed to detect and imagine miR-21 and H_2_O_2_ in a simulated ischemia reperfusion injury in both living cells and in vivo. H_2_O_2_ regulated miRNA-21 via the PI3K/AKT signaling pathway for the first time in H9C2 cells in a simulated ischemia reperfusion injury. All the results revealed that there was definitely a cROS talk between miR-21 and H_2_O_2_ in ischemia reperfusion injuries, and the current technique could explore the interplaying roles between miRNAs and ROS in various pathological procedures [133]. 

More recently, electrospun PCL and PCL-gelatin blend (PCLG) nanofibers were decorated with CNPs to prepare an antioxidant and antihypertrophic cardiac patch [134]. The prepared nanofibers showed good compatibility with fibroblasts, myotubes, and cardiomyocytes, as well as could alleviate agonist-induced hypertrophy in primary cardiomyocytes in vitro, thanks to the scavenging ROS activity of nanoceria. 

Regarding the literature, there are still huge opportunities and challenges in the case of CNP-containing constructs for cardiac tissue engineering applications; therefore, researchers and scientists are suggested to design innovative CNP-based formulations and evaluate their efficacy both in vitro and in vivo. Three-dimensional printed scaffolds may be excellent candidates to be incorporated with nanoceria for generating antioxidant cardiac tissue replacements.

### 4.4. Ophthalmic Applications

Various CNP-based therapeutic formulations have been developed for ophthalmic applications, including the treatments of both anterior and posterior eye segments, mainly due to the redox-active radical scavenging activities (antioxidant activity) of nanoceria [135,136]. 

#### 4.4.1. Ocular Surface Applications

**Figure 6:**
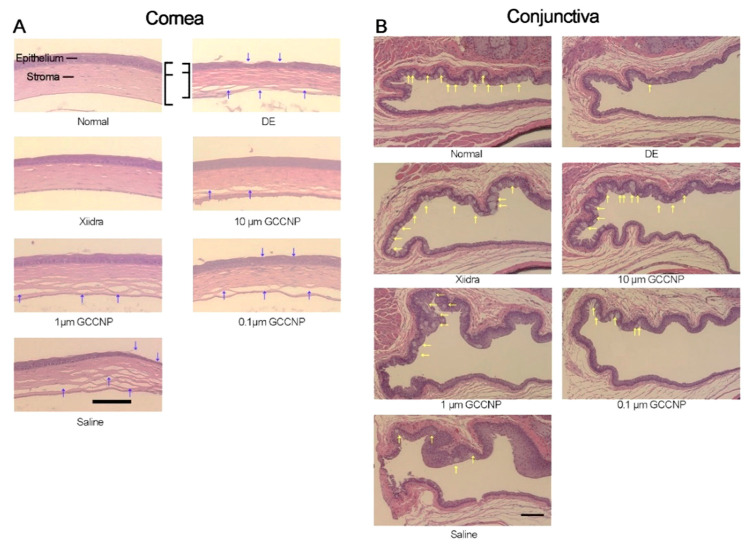
Histological micrographs taken from the cornea and conjunctiva of normal mice, mice with dry eye (DE), and mice that received different dosages (10, 1, and 0.1 μM) of CNP-loaded glycol chitosan (GC) nanocarriers (GCCNP) for 7 days. (**A**) Showing the corneal morphology (the blue arrows) and (**B**) exhibiting goblet cells in the conjunctiva (the yellow arrows). Scale bar: 200 μm. Reproduced with permission from [137].

Eye drops are among the most widely used therapies for ophthalmic disorders; Yu et al. recently developed a water-soluble CNP-loaded glycol chitosan (GC) nanocarrier (GCCNP) for potential use in managing dry eye (DE) disease [137]. The incorporation of nanoceria into GC led to increasing the cerium solubility from 0.020 ± 0.002 μg/mL to 709.854 ± 24.3 μg/mL. To evaluate the constructs’ efficacy, the authors used Lifitegrast (Xiidra), a Food and Drug Administration (FDA)-approved DE drug, as the positive control group. No toxicity was observed after the incubation of mouse corneal and conjunctival primary cultures with variable concentrations of GCCNP (0.1, 1, and 10 μM). In addition, the 1.0-μM and 10-μM GCCNP groups showed a significant inhibitory effect on ROS generation compared to the positive control, indicating a drastic scavenging activity of the GCCNP against intracellular ROS in corneal and conjunctival cells. This may be related to the ability of both cell types to uptake the GCCNPs that can reach the optimal location and clear excess ROS produced by mitochondria. As tear volume is an important index for assessing DE in the clinical setting, the GCCNP impact on tear volume changes in DE mice models was tested within seven days. The tear volumes in the DE group (0.42 ± 0.054 μL) were increased up to 0.47 ± 0.066, 0.56 ± and 0.14, 0.72 ± 0.15 μL in the case of 0.1, 1, and 10-μM GCCNP-treated groups, respectively. The best outcome belonged to the 10-μM GCCNP and Xiidra group (0.86 ± 0.18 μL), which showed a significant improvement. Histological evaluations clarified that the treatment with 10 μM of GCCNP and Xiidra is effective for the recovery of damaged corneal epithelial layers and returning corneal morphology to a nearly normal status, with a similar epithelial thickness and stromal morphology (see Figure 6). 

#### 4.4.2. Glaucoma Treatment

**Figure 7:**
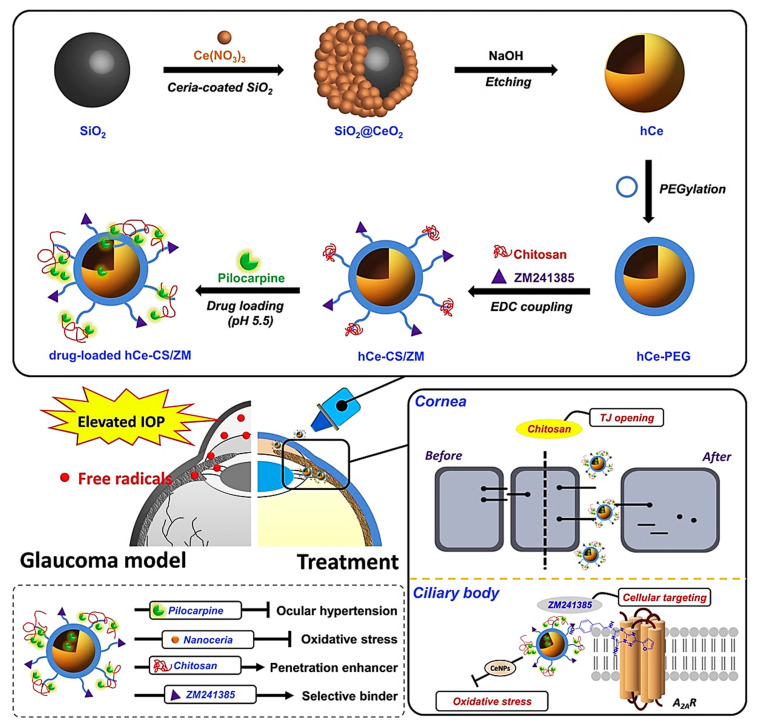
Schematic representation of the formulated nano-eye drops and their application for treating glaucoma. The silica templating method was used to synthesize hollow ceria nanoparticles (hCe NPs) and functionalize the samples with chitosan and ZM241385, which then were loaded with pilocarpine for the potential use as nano-eye drops (drug-loaded hCe-CS/ZM). Reproduced from [138].

In order to have an effective glaucoma treatment (especially in the case of chronic disease), it is essential to deliver ophthalmic drugs to the eye’s inner parts. This procedure still remains a big challenge due to dynamic and static ocular barriers. Thus, an ultimate drop nano-formulation in the treatment of glaucoma should have the necessary criteria, including the ability to penetrate the cornea and deliver bioactive molecules to target tissues, a high drug retention in ocular tissues, and sustained pharmacological activities. In this regard, Luo et al. recently fabricated a dual-functional hollow nanoceria platform as a new antiglaucoma formulation for the intraocular targeting and sustained delivery of pilocarpine [138]. The construct aimed to overcome the static and dynamic ocular barrier limitations by opening the corneal epithelial tight junctions. The system showed an additional therapeutic capacity for alleviating glaucomatous damage regarding the antioxidant and anti-inflammatory properties of CNPs. The authors used chitosan (CS) and ZM241385 (ZM) (a nonxanthine adenosine receptor antagonist binding to the A2AR subtype in the ciliary body tissue) for the surface functionalization of the hollow ceria nanoparticles (hCe NPs), enabling them to open corneal epithelial tight junctions and deliver pilocarpine to the targeted intraocular tissue (i.e., ciliary body) (See Figure 7). The cerium concentrations in the nano-drops were 9.6 ± 4.2, 63.1 ± 5.7, and 1.9 ± 0.3 μg/g in the hCe-CS1/ZM, hCe-CS2/ZM, and hCe-CS3/ZM groups, respectively. The sustained release of pilocarpine from the dually functional hCe NPs was recorded over seven days. In vivo evaluations were performed on a rabbit model of glaucoma; the one-time topical instillation of this nano-drop system resulted in a significant improvement in the attenuation of the experimental glaucoma progression, demonstrating a 42-fold longer period to lower the elevated intraocular pressure (IOP) to normal levels. 

#### 4.4.3. Retinal Applications

Several experimental studies have shown nanoceria’s usefulness in different formulations for treating retinal damage, such as acute damage due to the exposure to high-intensity light [139].

Furthermore, previous in vivo studies have shown that nanoceria could specifically prevent retina’s neurodegeneration for a long time, with no collateral effects [140]. The long-term safety of nanoceria in ophthalmic tissue was documented, as it had no negative influences on the function or cytoarchitecture of rat retinas [141]. Due to its nano-scaled diameters, the topical treatment of nanoceria could be attained via corneal permeation through PEGylation and liposomal encapsulation strategies, with no changes in the physicochemical properties of nanoceria, making it a potent candidate for treating several eye disorders in the posterior segment [142].

The most common ophthalmic drug delivery route to treat vitreoretinal diseases is intravitreal (IVT) injection to the eye posterior segment. This method was successfully employed by Wong et al. to deliver Alexa Fluor 647–conjugated nanoceria to the vitreous of both male and female BALB/c mice. A delay in disease progression was observed after a single IVT of inorganic antioxidant catalytic nanoceria, which confirmed nanoceria as a suitable ophthalmic carrier to the retina. Although they showed that their synthesized nanoceria could be retained in the retina for over a year, retina cell types that preferentially take up nanoceria are yet to be discovered [143].

#### 4.4.4. Contact Lenses

The use of therapeutic contact lenses with growing drug bioavailability has been widely investigated as a brilliant alternative for conventional treatment routes of eye diseases [144]. Although the common fabrication approaches of these drug-eluting contact lenses (e.g., molecular imprinting) provide successful continuous and controlled ocular drug delivery, their optical and physical performance can be improved by nano-implants. Moreover, the undesirable release of the loaded drug into the packing solution during the storage and delivery process requires developing an upgraded type of therapeutic contact lens to overcome these issues [145]. In a recent study by Choi et al., a water-soluble ROS scavenging nanoceria-embedded contact lens was developed to prevent ocular surface-related diseases. This nanoceria-containing lens demonstrated outstanding extracellular ROS scavenging properties and protective effects in a mouse model after the administration of 3% H_2_O_2_ eye drops, along with a high comparable transparency and physical properties to those of a commercial contact lens. The results of this study indicated that this novel nanoceria-containing lens could be useful in the treatment of ocular surface diseases [146].

#### 4.4.5. Crystalline Lens Applications

Based on a study by Pierscionek et al., dosages of 5 and 10 μg/mL of nanoceria caused no damage to the DNA, nor raised the chromosomal exchanges in cultured human crystalline lens epithelial cells [56]. Although low concentrations of nanoceria have presented protective effects on human lens epithelial cells against oxidative stress, the mechanism of its genotoxic and cytotoxic properties at higher concentrations has still been undiscovered. Hanafy and colleagues investigated the effect of a 24-h exposure to nanoceria on human crystalline lens epithelial cells. The effects of nanoceria on basal ROS, genotoxicity, membrane potential, mitochondrial morphology, ATP, apoptotic hallmarks, and caspase activation were also studied. According to their results, high concentrations of nanoceria (400 µg/mL) increased the intracellular levels of ROS, and the human lens epithelial cells revealed the classical hallmarks of apoptosis, which agrees with the cells retaining the normal ATP levels necessary to complete the apoptotic process. This study highlighted the necessity of focused studies on nanoceria dose-dependent effects on various cells and tissues to recognize therapeutic in vitro and in vivo concentrations [147]. 

## 5. Nanoceria: Remaining Issues before Clinical Trials

Early reports on the effectiveness of cerium in managing soft-tissue diseases and injuries date back to the 1970s [148,149]; the antibacterial property of cerium compounds (nitrate or sulphadiazine) was the main reason behind their use for treating infectious burn wounds [150]. Although the observations indicated the lack of major toxicological side effects of cerium compounds in damaged sites, dermal calcification raised some concerns regarding the extensive use of cerium in wound-healing applications [150]. Still, randomized controlled trials (RCTs) are being conducted to take benefit from cerium compounds in managing wounds (e.g., facial burns), and satisfactory outcomes were reported regarding both aesthetics and functionality [151]. 

Over the years, CeO_2_ nanoparticles were introduced as potentially effective substances for treating a wide range of soft tissue-related injuries. Compared to their coarser counterparts (i.e., microparticles), nanoceria exhibit unique features, including a large number of surface defects—primarily surface oxygen vacancies—providing the ability to switch oxidation states between III and IV and yielding antioxidant activity [152,153]. A lot of in vitro and in vivo animal studies have proven the therapeutic effectiveness of various sizes and shapes of CeO_2_ nanoparticles for treating soft-tissue injuries [154,155]; however, no clinical trials have been registered yet in which ceria nanoparticles are utilized for treating human diseases. The reasons may be connected with the lack of necessary guidelines and care protocols for nanoceria clinical use. In this regard, biomedical scientists are suggested to carefully design RCTs for showing the actual therapeutic impact of nanoceria, either alone or as formulated with other substances, in managing both acute and chronic wounds. Before that, more research is required to fundamentally evaluate the accumulation and clearance of nanoceria of target tissues/organs (e.g., skin, heart, and so on) and the other vital organs (e.g., the kidneys), as well as to define the best administration dosage and route. In brief, cerium compounds have been practically used for treating soft-tissue wounds, but much caution should be applied when using nanoceria, as their pharmacokinetics and distribution are quite different than their bulk counterparts.

## 6. Concluding Remarks

Nanoceria has been convincingly proven to exhibit appealing properties for applications in contact with a range of soft tissues. The most significant properties of nanoceria include proangiogenic activity, which is the key to accelerating wound healing and is also effective in the treatment of diabetic chronic wounds, and radical scavenging activity, which is behind its antioxidant properties and plays a pivotal role in the therapy of some neural, cardiac, and ocular diseases. These beneficial biological effects are mainly dictated by the nano-formulation of cerium oxide, which, on the other hand, is an inherent limitation of this material. In fact, nanoceria suffers from similar safety-related drawbacks of other nonsoluble nanomaterials, the toxicity of which depends on the shape and dosage of the nanoparticles. The incorporation of nanoceria in polymeric matrices (e.g., 3D scaffolds) or coatings with polymers are possible options to improve the biocompatibility but might concurrently decrease the therapeutic effect. 

The current literature witnesses that CNPs can overcome some physiological barriers that instead limit the diffusion of organic drugs into some districts/organs of the body, like the brain and posterior segment of the eye, which are often challenging to treat pharmacologically. This, indeed, motivates further research on this material and, also, suggests the need for a selection of the most promising applications, where nanoceria can carry a clear, invaluable added value compared to the existing alternatives. Future studies should be addressed to elucidate the fate of CNPs in the long term and quantify the minimum therapeutic concentrations of nanoceria that can be administered to different target tissues without eliciting toxic effects. The implementation of 3D printing strategies could also be useful to properly design the structure of the implants in which CNPs are incorporated in the attempt to finely tune the nanoceria release for performing the best therapeutic actions.
